# 

**Published:** 1893-12

**Authors:** 


### CONTENTS.

PROCEEDINGS.

World's Columbian Dental Congress—1893.............................. 573

The Southern Dental Association—1893.............................. 612

Alumni Meeting—University of Michigan.............................. 614

EDITORIAL.

Food .............................................................. 615

Bibliography ...................................................... 616

